# Influence of reoxygenation at different temperatures on growth, hematological and oxidative stress parameters in *Piaractus mesopotamicus*

**DOI:** 10.1007/s10695-026-01715-y

**Published:** 2026-06-05

**Authors:** Luana Gabrieli Lamberti, Lilian Fiori Nitz, Liliane Soares Presa, Lucas Pellegrin, Andressa Larré Bitencourt, Luciano Garcia

**Affiliations:** 1 Laboratório de Aquacultura Continental, Instituto de Oceanografia, Universidade Federal do Rio Grande - FURG, Rio Grande, RS 96203-900 Brazil; 2 Programa de Pós Graduação em Aquicultura, Universidade Federal do Rio Grande - FURG, Rio Grande, RS Brazil

**Keywords:** Hypoxia, Freshwater fish, Erythrocytes, Hemoglobin, Total antioxidant capacity, Lipid peroxidation

## Abstract

This study aimed to evaluate the effects of the interaction between different temperatures and reoxygenation rates on growth performance, as well as hematological and oxidative stress parameters in pacu (*Piaractus mesopotamicus*) juveniles. The animals were exposed to 8 h of severe hypoxia on the 15th day and 30th day (0.8 mg L^−1^) followed by abrupt reoxygenation or gradual, at two temperatures (24.11 ± 0.10 and 27.08 ± 0.05 ºC). A control treatment was established for each temperature, maintained at normoxia (≥ 7.0 mg L^−1^) throughout the experimental period (45 days). Final biometry was performed, as well as the collection of blood and tissues (gills, brain, liver, and muscle) to determine growth performance, hematological parameters, total antioxidant capacity against peroxyl radicals (ACAP), and lipid peroxidation (LPO). Results showed that the growth parameters were higher at 27 ºC. At this temperature, the animals showed higher levels of hyperglycemia compared to juveniles kept at 24 ºC. Fish subjected to different reoxygenation rates at 27 ºC showed a reduction in hemoglobin and the number of erythrocytes, in addition to a higher mean corpuscular volume (MCV) compared to the normoxic treatment at the same temperature and animals exposed to the same reoxygenation rates at 24 ºC. Lower ACAP levels were observed in the liver and muscle of fish kept at 24 ºC compared to animals kept at 27 ºC. Higher LPO levels in all tissues were induced by the interaction of 24 ºC with normoxia and gradual reoxygenation, and 27 ºC with abrupt reoxygenation. In conclusion, the growth of the species is optimized at 27 ºC. Moreover, at this temperature, the animals present better physiological conditions in the face of gradual reoxygenation and can be considered tolerant to hypoxia and reoxygenation when exposed to this situation in the farming environment.

## Introduction

Pacu (*Piaractus mesopotamicus*) is found in the basins of the Paraná, Paraguay, and Uruguay rivers (Godoy 1975). Its cultivation is of great importance in South American countries such as Brazil, Argentina and Paraguay (Valladão et al. 2016). This specie can serve as model of species resistant to different water quality parameters in aquatic organism farming environments. Therefore, its farming in confined environments, at high density and different production systems (RAS, BFT, symbiotic systems) is viable, due to showed a fast growth rate, efficient feed conversion, rusticity, and good fillet quality. In addition, pacu has a high tolerance to low levels of dissolved oxygen (Nitz et al. 2020a), wide temperature ranges (Pinto et al. 2020), different pH levels (Copatti et al. 2019a), ammonia (Nitz et al. 2019) and nitrate (Fortunato et al. 2024), which demonstrates the promising potential for the cultivation of the species in intensive systems (Pellegrin et al. 2022).

In aquaculture systems, variations in dissolved oxygen (DO) concentration are common, since this depends on mechanical aerators, which are susceptible to problems such as lack of energy (Dong et al. 2011; Zhan et al. 2023), stocking density (Bittencourt et al. 2010), and concentration of decomposing organic matter that consumes DO from the environment.

Extreme oxygen fluctuations, as well as hypoxia, can be frequent and may lead to reductions in food consumption, growth rate, and fish survival (Magnoni et al. 2018; Marcek et al. 2020; Jiang, et al. 2023). Hypoxia also affects physiological and biochemical responses, including hematological responses (Moraes et al. 2002; Dillhon et al. 2018; Jia et al. 2021; Witeska et al. 2022; Ranzani-Paiva and Tavares-Dias 2024) and oxidative stress (Copatti et al. 2019b; Nitz et al. 2020a; Refaey et al. 2025). However, although hypoxia represents a major challenge to fish survival and well-being, subsequent reoxygenation can be even more stressful, especially due to the large production of reactive oxygen species (Lushchak and Bagnyukova 2006a; Ali et al. 2012; Zhan et al. 2023; Cao et al. 2025).

Oxidative stress is defined as an imbalance between reactive oxygen species (ROS) production and the body's antioxidant defenses. It can cause DNA hydroxylation, protein denaturation, lipid peroxidation, apoptosis, and cellular damage (Martínez-Álvarez et al. 2005; Shuang et al. 2023).

The antioxidant defense system is divided into two groups: (i) enzymatic, composed of enzymes synthesized by the body and (ii) non-enzymatic (Wang et al. 2021). The activity of antioxidant enzymes is related to the metabolic rate of organisms and, therefore, is subject to the influence of temperature (Welker et al. 2013; Wang et al. 2021; Shuang et al. 2023).

For most fish species, growth is optimized in a narrow temperature range; therefore, exposing animals to temperatures outside this ideal range can induce a pro-oxidant state (Feng et al. 2012; Vinagre et al. 2012; Pinto et al. 2020). The optimal temperature range for pacu cultivation is between 23 and 29 ºC (Garcia et al. 2008; Pinto et al. 2020).

This study aimed to evaluate the effects of the interaction between different temperatures (24 and 27 ºC) and two reoxygenation rates: abrupt (normoxia levels restored within 20 min) or gradual (normoxia levels restored within 5 h), after 8 h of hypoxia on blood, growth performance, and oxidative stress parameters in pacu juveniles after 45 days.

## Material and methods

### Animal ethics

The experimental protocol for this study was approved by the Comitê de Ética e Uso Animal (CEUA) of the Federal University of Rio Grande – FURG, protocol number P025/2021.

### Animals, acclimatization, and experiment

Two hundred and sixteen pacu juveniles (15.79 ± 0.16 g; 9.14 ± 0.03 cm) were obtained from a commercial fish farm in Victor Graeff, Rio Grande do Sul, Southern Brazil. The fish were kept in a recirculation system with 310 L tanks (250 L of useful volume) before the experiment started. The animals were fed twice daily (9 AM and 4 PM), with a diet containing 32% crude protein, until apparent satiety. The same feeding protocol was followed during the acclimatization period to the experimental conditions. Feeding was suspended throughout hypoxia.

Prior to the experiment, the animals were randomly distributed into 18 tanks (80 L of useful volume) and acclimated to temperatures of 24 and 27 ºC, in six recirculation systems with mechanical and biological filters, constant aeration and a photoperiod fixed at 9L/15D (Presa et al. 2024) for 10 days.

The water temperature of the experimental units (EU) was adjusted with air conditioning at 24 ºC and heaters with thermostats at 27 ºC. The temperatures were chosen according to Pinto et al. (2020; 2022) for the species.

The maintenance of DO levels in normoxia (7.40 ± 0.02 mg L^−1^) was carried out using an air blower (1 HP) that supplied the EU individually through the aeration system. Over the 45 experimental days, the animals were maintained in normoxia except in two periods: on the 15th (0.83 ± 0.07 mg L^−1^) and 30th day (0.84 ± 0.01 mg L^−1^), where they were subjected to hypoxic conditions for 8 h at two different temperatures (24.11 ± 0.10 ºC and 27.08 ± 0.05 ºC). Pure nitrogen gas was injected through individual diffusers into each tank until the desired levels of hypoxia were obtained. These two hypoxia periods were used to simulate a lack of energy during the production period.

Before initiating the nitrogen injection into the water of the EU, the aeration system was turned off. Once hypoxia levels were reached, they were maintained through constant monitoring (every 1 h) using a digital oximeter (YSI® 200 A).

After the period of hypoxic exposure, two types of reoxygenation rates were immediately performed: abrupt, in which the normoxic condition was reestablished within 20 min; and gradual, where the DO concentration was gradually increased by 1 mg/L/h over 5 h. During gradual reoxygenation, oxygen saturation was monitored continuously using a digital oximeter. All reoxygenation rate processes were performed following Nitz et al. (2020b).

A control treatment, maintained in normoxia (≥ 7.0 mg L^−1^), without exposure to hypoxia or reoxygenation, was subjected to the same temperatures. The experimental protocol included six randomized treatments in triplicate (*n* = 12 fish per replicate).

### Water quality

Water quality parameters were maintained at desirable levels for the species during the acclimatization and experimentation period—except for DO, which was changed for the experiment. Water physicochemical variables, temperature (24.11 ± 0.10 ºC; 27.08 ± 0.05 ºC) and DO (7.40 ± 0.02 mg L^−1^; YSI® 200 A oximeter), pH (8.03 ± 0.09; Hanna® HI8424 pH meter), total ammonia (0.18 ± 0.003 mg N-NH_3_ L^−1^; UNESCO 1983), nitrite (0.12 ± 0.02 mg N-NO_2_ L^−1^; Boyd and Tucker 2014), and alkalinity (66.17 ± 4.80 mg CaCO_3_ L^−1^; Eaton et al. 2005) were monitored daily.

### Zootechnical performance

Biometric measurements were taken on all animals at the beginning (day 0) and the end of the experimental period (day 45) to determine zootechnical performance. For this procedure to be carried out, the fish were removed from the tank and anaesthetized with benzocaine hydrochloride (50 ppm). The following calculations were performed:

$$Final\mathit\;average\mathit\;weight\mathit\;\mathit(FW\mathit,\mathit\;g\mathit)\mathit\;\mathit=\mathit\;\mathit\sum\mathit\;final\mathit\;weight\mathit\;of\mathit\;live\mathit\;animals\mathit\;\mathit(g\mathit)\mathit\;\mathit/\mathit\;total\mathit\;number\mathit\;of\mathit\;animals$$

$$Average\mathit\;final\mathit\;total\mathit\;length\mathit\;\mathit(TL\mathit,\mathit\;cm\mathit)\mathit\;\mathit=\mathit\;\mathit\sum\mathit\;final\mathit\;total\mathit\;length\mathit\;of\mathit\;live\mathit\;animals\mathit\;\mathit(cm\mathit)\mathit\;\mathit/\mathit\;total\mathit\;number\mathit\;of\mathit\;animals$$

$$Weight\mathit\;gain\mathit\;\mathit(WG\mathit,\mathit\;g\mathit)\mathit\;\mathit=\mathit\;final\mathit\;body\mathit\;weight\mathit\;\mathit(g\mathit)\mathit\;\mathit-\mathit\;initial\mathit\;body\mathit\;weight\mathit\;\mathit(g\mathit)$$

$$Specific\;growth\;rate\;(SGR,\;\%\;per\;day)\;=\;\mathit{100}\;\times\;(Ln\;final\;weight\;(g)\;-\;Ln\;initial\;weight\;(g))\;/\;time\;(days)\;$$

$$Condition\mathit\;factor\mathit\;\mathit(CF\mathit,\mathit\;g\mathit\;cm^{\mathit-\mathit3}\mathit)\mathit\;\mathit=\mathit\;\mathit{100}\mathit\;\mathit\times\mathit\;\mathit(body\mathit\;weight\mathit\;\mathit(g\mathit)\mathit/body\mathit\;length\mathit\;{\mathit(cm\mathit)\mathit)}^{\mathit3}$$

$$Survival\mathit\;\mathit(\mathit\%\mathit)\mathit\;\mathit=\mathit\;\mathit{100}\mathit\;\mathit\times\mathit\;\mathit(final\mathit\;fish\mathit\;number/initial\mathit\;fish\mathit\;number\mathit)$$

### Tissue collection

After biometric procedures (45 days), three anesthetized fish per tank (*n* = 9 per treatment) were randomly sampled for whole blood collection (1.5 mL) from the caudal vasculature using heparinized (Hepamax®) syringes. Blood was collected from anesthetized animals (benzocaine hydrochloride 50 ppm). Immediately after whole blood collection, these animals were euthanized with a lethal dose of benzocaine hydrochloride (500 ppm) for tissue collection.

Gill, brain, liver, and muscle samples were collected and stored in 2 mL microtubes, frozen in liquid nitrogen, and kept in an ultrafreezer at −80ºC until analysis.

### Hematological parameters

Blood glucose levels were analyzed using a digital glucometer (Accu-check Performa/Roche®), and blood pH was measured using a pH meter (HANNA HI 2210®), with a probe designed for this purpose. Hematocrit (Hct) was determined by centrifuging heparinized capillary tubes containing a blood aliquot at 12,000g for 5 min (Goldenfarb et al. 1971). Hemoglobin (Hb) concentration was then measured using a colorimetric kit (Labtest®) with a spectrophotometer at 540 nm. An aliquot of collected blood was diluted at a ratio of 1:200 in a 0.65% sodium chloride solution to achieve the appropriate concentration for counting erythrocytes. The erythrocyte concentration was then determined using a Neubauer chamber and an optical microscope (Ranzani-Paiva et al. 2024). All blood tests were performed immediately after collection. Hematimetric indices (MCV, MCH and MCHC) were estimated according to Pinto et al. (2020).

### Tissue homogenization

Tissue samples were homogenized (1:5, m/v) in a Tris–HCl buffer (100 mM, pH 7.75) containing EDTA (2 mM) and Mg^2+^ (5 mM) (Da Rocha et al. 2009), centrifuged (10,000 g, 20 min, 4ºC), and the supernatants were used for all analyses. Total protein content of the homogenates was determined by the Biuret method using a commercial kit (Doles®). Total antioxidant capacity against peroxyl radicals (ACAP) was determined according to the Amado et al. (2009). Lipid peroxidation (LPO) levels were determined according to Oakes and Van Der Kraak (2003).

### Statistical analysis

Treatments were analyzed in triplicate. All results are expressed as mean ± standard error. The normality of the data and the homogeneity of the variances were previously tested using the Kolmogorov–Smirnov and Levene tests, respectively. Data were subjected to two-way ANOVA (temperature x DO or reoxygenation), followed by a Tukey post-hoc test. The analyses were performed with a minimum significance level of *p* < 0.05.

## Results

### Zootechnical performance

No mortality was observed during the acclimatization and experimentation periods. The animals kept at a temperature of 27 °C showed significantly higher growth performance indices (mean final weight (FW, g), mean final total length (TL, cm), weight gain (WG, g), and specific growth rate (SGR, % per day)) (*p* < 0.05) when compared to fish exposed to 24 °C. However, the body condition factor (FC, g cm^−3^) showed no significant difference (*p* > 0.05) between temperatures. Juveniles in the normoxia and 27 ºC treatments presented a significantly higher body condition factor (*p* < 0.05) compared to the abrupt reoxygenation treatment (Table 1).

**Table 1:** Results of zootechnical performance and survival of pacu juveniles (*P. mesopotamicus*) exposed to the interaction between different temperatures and abrupt (20 min) or gradual (5 h) reoxygenation, or kept only under normoxia (7.40 mg L^−1^) for 45 days

Variables	Temperature					
	24 ºC			27 ºC		
	Normoxia	Abrupt	Gradual	Normoxia	Abrupt	Gradual
FW (g)	31.84 ± 1.05^Ba^	35.01 ± 1.24^Ba^	32.74 ± 1.14^Ba^	53.00 ± 2.03^Aa^	49.89 ± 1.68^Aa^	52.03 ± 1.61^Aa^
TL (cm)	10.96 ± 0.11^Ba^	11.24 ± 0.16^Ba^	11.19 ± 0.13^Ba^	13.00 ± 0.18^Aa^	12.96 ± 0.15^Aa^	13.05 ± 0.16^Aa^
WG (g)	16.94 ± 0.94^Ba^	17.78 ± 1.28^Ba^	17.53 ± 1.06^Ba^	38.55 ± 2.25^Aa^	33.08 ± 1.82^Aa^	35.86 ± 1.69^Aa^
SGR (% per day)	37.65 ± 2.10^Ba^	39.51 ± 2.85^Ba^	38.95 ± 2.36^Ba^	85.67 ± 4.52^Aa^	73.52 ± 4.05^Aa^	79.69 ± 3.76^Aa^
CF (g cm^−3^)	2.37 ± 0.03^Aa^	2.39 ± 0.05^Aa^	2.33 ± 0.04^Aa^	2.39 ± 0.01^Aa^	2.31 ± 0.01^Ab^	2.33 ± 0.03^Aab^
S (%)	100	100	100	100	100	100

Data are expressed as mean ± SE (*n* = 36). Different capital letters indicate significant differences between temperatures at the same reoxygenation rate (*p* < 0.05). Different lowercase letters indicate significant differences between reoxygenation rates at the same temperature (*p* < 0.05). Abbreviations: *FW* final weight, *TL* total length, *WG* weight gain, *SGR* specific growth rate, *CF* body condition factor, *S* survival

### Hematological parameters

Blood glucose levels were significantly higher (*p* < 0.05) in fish kept at 27 ºC compared to those kept at 24 ºC at the same reoxygenation rate or normoxia. The number of erythrocytes was significantly higher (*p* < 0.05) in animals treated with normoxia at 27 °C compared with fish exposed to abrupt and gradual reoxygenation at the same temperature. Animals submitted to abrupt and gradual reoxygenation also at 27 ºC presented significantly lower Hb concentrations (*p* < 0.05) compared to those kept in normoxia at the same temperature. The Hb concentration of juveniles in the normoxia treatment at 27 ºC was significantly higher (*p* < 0.05) compared to fish in the same treatment at 24 ºC (Table 2).

**Table 2:** Hematological parameters of pacu juveniles (*P. mesopotamicus*) exposed to the interaction between different temperatures and abrupt (20 min) or gradual (5 h) reoxygenation, or maintained only under normoxia (7.40 mg L^−1^) and for 45 days

Variables	Temperature					
	24 ºC			27 ºC		
	Normoxia	Abrupt	Gradual	Normoxia	Abrupt	Gradual
Glu	63.67 ± 1.83^Ba^	67.22 ± 1.47^Ba^	69.33 ± 1.93^Ba^	72.78 ± 3.13^Aa^	77.22 ± 3.42^Aa^	78.00 ± 3.28^Aa^
pH	7.42 ± 0.03^Aa^	7.51 ± 0.02^Aa^	7.49 ± 0.04^Aa^	7.47 ± 0.02^Aa^	7.52 ± 0.03^Aa^	7.52 ± 0.03^Aa^
Htc	27.44 ± 1.23^Aa^	26.56 ± 1.72^Aa^	27.56 ± 1.75^Aa^	29.33 ± 1.85^Aa^	30.33 ± 1.80^Aa^	29.67 ± 1.87^Aa^
Eri	1.75 ± 0.09^Aa^	1.57 ± 0.09^Aa^	1.54 ± 0.14^Aa^	1.70 ± 0.04^Aa^	1.51 ± 0.04^Ab^	1.41 ± 0.07^Ab^
Hb	7.80 ± 0.12^Ba^	7.64 ± 0.15^Aa^	7.71 ± 0.21^Aa^	8.63 ± 0.20^Aa^	7.19 ± 0.19^Ab^	7.59 ± 0.23^Ab^
MCV	169.03 ± 5.69^Aa^	160.64 ± 7.66^Ba^	170.48 ± 10.41^Ba^	179.20 ± 6.47^Ab^	194.55 ± 6.92^Aab^	209.80 ± 8.41^Aa^
MCH	45.37 ± 2.38^Aa^	45.76 ± 1.21^Aa^	50.85 ± 2.40^Aa^	50.97 ± 1.51^Aa^	47.61 ± 1.10^Aa^	52.84 ± 1.83^Aa^
MCHC	29.04 ± 1.81^Aa^	28.71 ± 1.59^Aa^	27.95 ± 1.99^Aa^	30.32 ± 2.01^Aa^	24.48 ± 1.83^Aa^	26.31 ± 1.67^Aa^

Data are expressed as mean ± SE (*n* = 9). Different capital letters indicate significant differences between temperatures at the same reoxygenation rate (*p* < 0.05). Different lowercase letters indicate significant differences between reoxygenation rates at the same temperature (*p* < 0.05). Abbreviations: *Glu* Glucose (mg L^−1^), *Hct* hematocrit (%), *Ery* erythrocytes (10^6^ µL^−1^), *Hb* hemoglobin (g dL^−1^), *MCV* mean corpuscular volume (fL), *MCH* mean corpuscular hemoglobin (pg), *MCHC* mean corpuscular hemoglobin concentration (g dL^−1^)

At a temperature of 27 ºC, the fish presented significantly higher levels (*p* < 0.05) of MCV compared to those at 24 ºC, at the same reoxygenation rate. The normoxia treatment at 27 ºC presented significantly lower MCV (*p* < 0.05) compared to the animals kept at the same temperature and subjected to gradual reoxygenation (Table 2).

### Total antioxidant capacity against peroxyl radicals (ACAP)

**Fig. 1:**
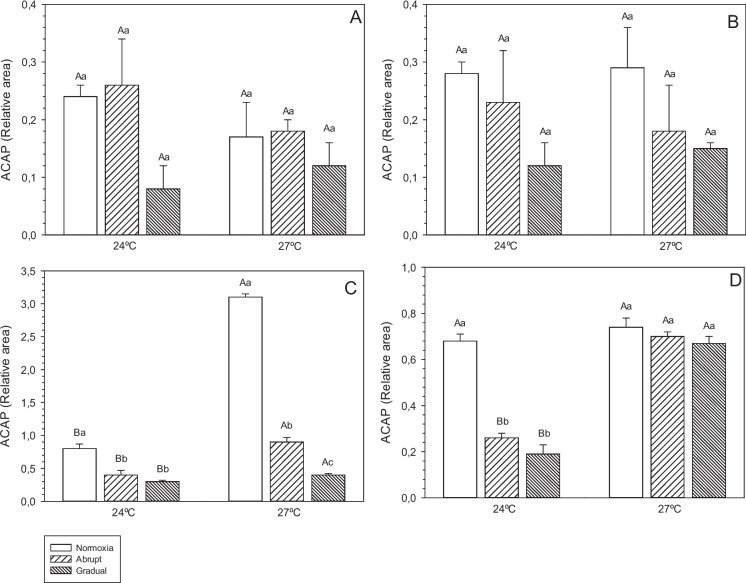
Total antioxidant capacity against peroxyl radicals (ACAP) in the gills (**A**), brain (**B**), liver (**C**) and muscle (**D**) of pacu juveniles (*P. mesopotamicus*) exposed to the interaction between different temperatures (24 and 27 ºC) and abrupt (20 min) or gradual (5 h) reoxygenation, or maintained only under normoxia (7.40 mg L.^−1^), for 45 days. Results expressed as mean ± SEM (*n* = 9). Different capital letters indicate significant differences between temperatures at the same reoxygenation rate (*p* < 0.05). Different lowercase letters indicate significant differences between reoxygenation rates at the same temperature (*p* < 0.05)

In general, ACAP levels were significantly lower (i.e., larger relative area; *p* < 0.05) in the liver and muscle of pacu juveniles maintained at 27 °C compared to those at 24 °C (Fig. 1C, D, respectively). Furthermore, at 27 °C, all treatments differed from one another in the liver (*p* < 0.05; Fig. 1C), with the normoxia treatment showing the largest relative area.

Fish submitted to different reoxygenation rates (abrupt and gradual) showed significantly higher levels of ACAP (smaller relative area) (*p* < 0.05) in the liver and muscle at 24 ºC, when compared with the normoxia treatment at the same temperature.

### Lipid peroxidation (LPO)

**Fig. 2:**
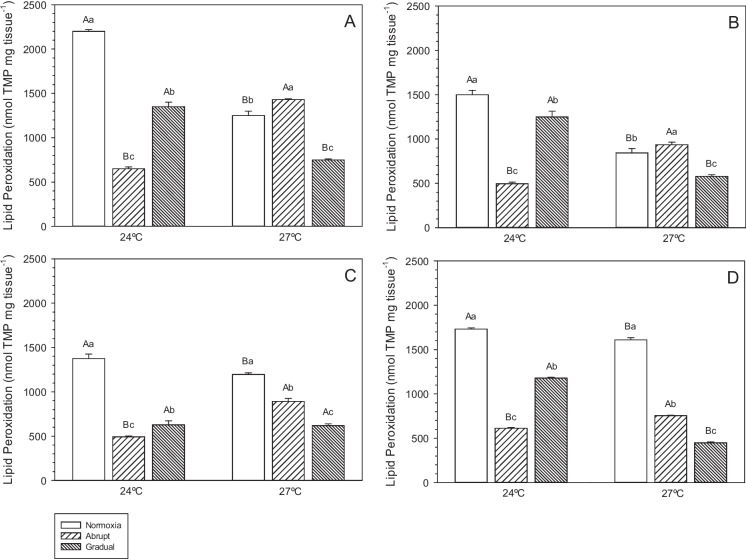
Lipid peroxidation (LPO) in the gills (**A**), brain (**B**), liver (**C**) and muscle (**D**) of pacu juveniles (*P. mesopotamicus*) exposed to the interaction between different temperatures (24 and 27 ºC) and abrupt (20 min) or gradual (5 h) reoxygenation or maintained only under normoxia (7.40 mg L.^−1^), for 45 days. Results are expressed as mean ± SEM (*n* = 9). Different capital letters indicate significant differences between temperatures at the same reoxygenation rate (*p* < 0.05). Different lowercase letters indicate significant differences between reoxygenation rates at the same temperature (*p* < 0.05)

Lipid Peroxidation levels were significantly higher (*p* < 0.05) in the different tissues (Fig. 2) of animals kept at 24 ºC compared to animals maintained at 27 ºC. However, in the abrupt reoxygenation treatment, LPO levels were significantly lower (*p* < 0.05) in animals exposed to 24 ºC.

At 24 ºC, LPO levels were highest under normoxia (*p* < 0.05), followed by the gradual reoxygenation, which resulted in intermediate levels across all tissues (*p* < 0.05; Fig. 2). The lowest levels (*p* < 0.05) of LPO were observed in the tissues of pacu juveniles submitted to gradual reoxygenation at this temperature.

At 27 °C, abrupt reoxygenation led to significantly higher LPO levels (*p* < 0.05) in the gills (Fig. 2A) and brain (Fig. 2B), while normoxia resulted in higher levels in the liver (Fig. 2C) and muscle (Fig. 2D). Fish subjected to gradual reoxygenation at 27 °C showed significantly lower LPO levels (*p* < 0.05) in all tissues compared to the other treatments at the same temperature.

## Discussion

Water temperature directly affects fish metabolic rates, affecting enzymatic activity and growth (Duan et al. 2017; Islam et al. 2019). Our study demonstrated that the growth performance indices of the species were optimized at 27 ºC, regardless of the treatment to which the animals were subjected. This result was expected, since the metabolism of ectothermic animals tends to accelerate at elevated temperatures, resulting in higher growth rates (Yang et al. 2018; Pinto et al. 2020). In these situations, fish may present changes in several metabolic parameters, mainly in blood glucose concentration (Martínez-Porchas et al. 2009; Refaey et al. 2025). This relation was evidenced in our study, in which the 27 ºC treatments simultaneously presented greater weight gain and glucose levels.

Temperature may act as a more effective glycemic inducer than factors associated with internal homeostasis (Polakof et al. 2012; Jia et al. 2024). Several authors report increased blood glucose levels in commercial fish species, such as *Horabagrus brachysoma* acclimated to 33 and 36 ºC (Dalvi et al. 2017), *Pangasianodon hypophthalmus* exposed to 36 ºC (Shahjahan et al. 2018), pacu kept at 30 ºC. (Pinto et al. 2019), and C*olossoma macropomum* and P*iaractus brachypomus* subjected to thermal shock (Souza et al. 2025). Thus, the higher glucose levels observed at 27 ºC in this study might be due to the influence of temperature on fish metabolic rate.

In aquaculture farming systems, episodes of hypoxia and subsequent reoxygenation are common and often result from factors such as high stocking densities, elevated temperature, and lack of energy (Bittencourt et al. 2010; Dong et al. 2011; Zhan et al. 2023). When exposed to such situations, fish increase the oxygen transport capacity of the blood by increasing the number of erythrocytes and hemoglobin-oxygen affinity, as well as decreasing MCHC, among others (Nikinmaa 2001; Wu et al. 2016; Jia et al. 2021; Souza et al. 2025). Previous studies have shown that primary hematological parameters such as erythrocyte number, Hb rate, and hematocrit increased in tambaqui (*Colossoma macropomum*, Affonso et al. 2002; Souza et al. 2025), sturgeon (*Acipenser schrenckii*, Ni et al. 2014) and Atlantic salmon (*Salmo salar*, Wood et al. 2019) during hypoxic conditions.

In contrast, this study showed a reduction in erythrocyte count in Hb concentration, as well as an increase in MCV in animals exposed to hypoxia and reoxygenation at 27 ºC compared to the normoxia treatment. This suggests a late adjustment to a possible hemolytic condition caused by the synergism between severe hypoxia (< 1 mg L^−1^) and reoxygenation (abrupt and/or gradual), since the lower number of erythrocytes and Hb concentration may indicate that hematopoiesis is occurring. This is evident due to the higher concentration of Hb in fish submitted to normoxia at 27 ºC, as in mature red blood cells, the concentration of this protein is higher (Bastos et al. 1977; Clark 1988). Furthermore, in fish previously subjected to stress, specifically severe hypoxia, the least physiologically costly response is an increase in cell volume, which indirectly enhances hemoglobin-oxygen affinity. As splenic recruitment of erythrocytes occurs, it results in a cardiovascular and hepatic overload and depletion of the red blood cell reservoir (Caldwell and Hinshaw 1994; Shuang et al. 2022, 2023). In the present study, this relationship was observed in animals exposed to stressors at 27 °C. Moreover, the lower MCV values ​​in animals exposed to reoxygenation at 24 °C can be explained by the greater availability of DO at lower temperatures (Witeska 2013).

In this study, the interaction between temperature, hypoxia, and both gradual and abrupt reoxygenation caused damage to the gills, brain, liver, and muscle of pacu juveniles at 24 and 27 ºC. Furthermore, alterations in total antioxidant capacity were observed in the liver and muscle, suggesting that the antioxidant defense system of these animals was affected by the interaction between temperature and reoxygenation rate. There was no mortality of pacu juveniles, proving that this species is tolerant to severe hypoxia and reoxygenation. The same results were shown in Nitz et al. (2020a) for the same specie.

Antioxidant capacity against peroxyl radicals (ACAP) is the set of enzymatic and non-enzymatic responses of the antioxidant defense system to neutralize oxidation and maintain the body's homeostasis (Amado et al. 2009; Pinto et al. 2019). Regardless of the temperature and reoxygenation rate or normoxia to which the pacu juveniles were exposed, no changes in ACAP in the gills and brain were observed in our study. These findings could indicate that SDA was able to neutralize long-term oxidative damage in these organs. In other study, total antioxidant capacity activity, in the branquial tissue of Chinese hook snout carp, exhibited a decreasing during hypoxia, and after occur the recovery to control levels during reoxygenation (Cao et al. 2025). However, an increase in LPO levels was observed in the gills and brain at 24 °C (normoxia and gradual) and 27 °C (normoxia and abrupt). These responses indicate that the antioxidant defense may have been insufficient to reduce ROS production in these tissues (Wang et al. 2021; Zhan et al. 2023), especially at 24 ºC, which presented the highest levels of damage in the different reoxygenations.

In general, LPO levels were higher in the gills, brain, and muscle of animals kept at 24 ºC, in normoxia and gradual reoxygenation treatments, compared to animals exposed to 27 ºC in the same treatments. ACAP levels were higher in the liver and muscle of pacu juveniles at 24 ºC, in all treatments except in the muscle of the normoxia treatment, compared to the same treatments at 27 ºC. This indicates that the SDA of these animals was less efficient in protecting against oxidative damage caused by the interaction between 24 ºC and reoxygenation rates. Temperature changes can cause alterations in the SDA response through the reduction of the activity of some antioxidant enzymes and depletion of the non-enzymatic defense system (Lushchak and Bagnyukova 2006b; Pinto et al. 2019), which corroborates our results.

At low temperatures, the metabolism of ectothermic animals is reduced, causing a decrease in food consumption and an increase in the degree of lipid unsaturation of biological membranes (Nitz et al. 2020a; Wang et al. 2023). Vinagre et al. (2012) observed that heat stress affects the antioxidant response of juvenile *Dicentrarchus labrax*, since LPO levels and catalase enzyme activity in the muscle were lower near the optimum temperature (24 ºC) and higher in fish exposed to temperatures outside the ideal range (above and below) for the species. Therefore, our findings indicate that 27 °C is the most suitable temperature for pacu cultivation as it promotes superior zootechnical performance indices, enhanced total antioxidant capacity, and lower lipid peroxidation levels when the fish are subjected to gradual reoxygenation.

Exposures to hypoxia trigger SDA responses, leading to a high risk of oxidative stress (Chowdhury and Saikia 2020). Furthermore, the reoxygenation process is also capable of stimulating the production of ROS in tissues and cells, resulting in oxidative stress and SDA disorder in fish (Lushchak et al. 2005; Lushchak and Bagnyukova 2006a; Refaey et al. 2025). This can be observed in the study by Lushchak et al. (2001), who found that LPO levels and GPx activity increased in the liver and brain of *Carassius auratus*, respectively, 14 h after rapid reoxygenation (return to normoxic levels within 30 min). GPx activity (liver) of common carp, in hypoxia and reoxygenation, does not change and and Chinese hook snout carp showed na increased under hypoxia conditions (Cao et al. 2025). In another study, Zhang et al. (2016), found an increase in MDA and LPO levels in the liver of *Pelteobagrus vachelli* after 4 and 6.5 h of hypoxic recovery.

In this study, we verified changes in the total antioxidant capacity in the liver and muscle, as well as high levels of LPO in the four analyzed tissues of pacu juveniles after 15 days of gradual and abrupt reoxygenation at 24 ºC and 27 ºC, respectively. These results indicate that the SDA of pacu juveniles was active in response to stress caused by reoxygenation rates at different temperatures. Similar results were found by Shuang et al. (2023), who observed an increase in SOD enzyme activity and MDA content in the gills of *Megalobrama amblycephala* after seven days of reoxygenation. Our study demonstrated that the most affected tissues (gills, brain, and muscle) exhibited tissue damage due to excess ROS, which was caused by gradual reoxygenation at 24 ºC and abrupt reoxygenation at 27 ºC. The same results were observed by Sun et al. (2020) in the stomach and intestine of *Lateolabrax maculatus*.

In our study, the responses to oxidative stress were organ-specific, since the four tissues presented distinct levels of LPO and ACAP at both reoxygenation rates. These results indicate that the organ-specific response is linked to the metabolic activity of each organ and, consequently, to its characteristic antioxidant defenses (Lushchak and Bagnyukova 2007).

The same response pattern was observed by Nitz et al. (2020a) for the same species when subjected to different temperatures and dissolved oxygen levels. However, the gills and brain showed good antioxidant capacity. Lipid peroxidation was increased in these tissues when subjected to gradual and/or abrupt reoxygenation at 24 and 27 °C, respectively. A similar response was observed in the liver of animals exposed to abrupt and gradual reoxygenation at 24 °C, as well as to gradual reoxygenation at 27 °C. However, in this case, a reduction in ACAP was observed at both temperatures, accompanied by higher levels of LPO in this tissue at the same reoxygenation rates, with the exception of abrupt reoxygenation at 24 °C. In muscle, despite the lower ACAP value at different reoxygenation rates at 27 ºC, LPO levels were reduced in normoxia and gradual reoxygenation treatments compared to animals at the same rates kept at 24 ºC.

In summary, this study showed that situations of hypoxia and reoxygenation gradual and abrupt at 24 and 27 ºC, respectively, induce lipid peroxidation in the tissues of pacu juveniles in the long term.

## Conclusion

In conclusion, pacu juveniles exhibit optimized growth rates when reared at 27 °C. Exposure to different temperatures and reoxygenation rates alters antioxidant capacity and lipid peroxidation. The interaction between severe hypoxia with 24 ºC and gradual reoxygenation, as well as with 27 ºC and abrupt reoxygenation led to oxidative damage. However, fish subjected to gradual reoxygenation at 27 ºC showed a greater ability to maintain antioxidant defenses and reduce lipid damage.

## Data Availability

The corresponding authors data supporting this study’s findings are available upon reasonable request.
